# Correlation of resin composite translucency and IOS accuracy: An *in-vitro* study

**DOI:** 10.4317/jced.61620

**Published:** 2024-06-01

**Authors:** Nam-Cong-Nhat Huynh, Anh-Thi-Van Tran, Thu-Nguyen-Trang Truong, Yen-Thi Le, Nguyen-Chi Tran, Trang-Thi-Ngoc Tran, Ding-Han Wang, Ming-Lun Hsu

**Affiliations:** 1 Faculty of Odonto-Stomatology, University of Medicine and Pharmacy at Ho Chi Minh City, Ho Chi Minh City, 749000, Vietnam; 2 Nikkori Dental Clinic, Ho Chi Minh City, 749000, Vietnam; 3 School of Dentistry, National Yang-Ming Chiao Tung University, Taipei, 112304, Taiwan

## Abstract

**Background:**

Different restoration materials have different optical characteristics that influence the intraoral scanner’s (IOS) image accuracy. The purpose of this *in-vitro* investigation was to investigate how composite translucency affected the accuracy of IOS.

**Material and Methods:**

GC G-aenial Universal Injectable JE composite plates were used for the study at 3 thicknesses (1-2-3mm). A lab scanner (3Shape E1) obtained 1 reference scan, whereas IOS (Trios3) was used to conduct 10 experimental scans per group. After 3D superimposition, deviation values were used to assess the accuracy (trueness and precision) outcomes for the corresponding groups. Using an LS170 V2.0 colorimeter, the translucency parameter (TP) of the plates was determined from L*a*b* values of CIELAB color space.

**Results:**

The composite translucency resulted in a decrease in the scale of digital impressions. The 1mm group had the largest scale reduction (0.02mm) significantly, followed by the 2mm and 3mm groups (0.01mm). No difference was found in mean precision. The colorimeter detects the L*a*b* values and showed that 1mm composite plate expressed the highest TP value, then 2mm and 3mm groups (28.90, 14.26 and 6.49 respectively). The thinner composite, the higher translucency and TP were highly positively correlated to IOS trueness of composite plates.

**Conclusions:**

Composite translucency has an impact on optical impression accuracy. In correlation, the optical impression becomes less accurate the more translucent the composite is. This implies that in the digital process, the dentist should specify the appropriate optical properties of composite materials concerning both their mechanical and aesthetic qualities.

** Key words:**Accuracy, translucency, resin composite, digital dentistry, intraoral-scanner.

## Introduction

Introduced around 1954, dental resin composite has come a long way in nearly 70 years of continuous improvements to become one of the most commonly used regenerative materials in dentistry for its unique properties: appropriate physical-mechanical, thermal properties, biocompatibility and aesthetics (1). In particular, aesthetics is the top concern of both patients and dentists. One of the factors influencing the aesthetics of the final restoration is the optical properties of the material, in which composite translucency plays an important role. Translucency is the ability of a material to allow light to pass through and allow the hue of the underlying background to show through. Composite translucency is often evaluated by the translucency parameter (TP) and this index varies between shades of the same composite (2). Through the spectrophotometer, the data obtained from measuring the amount of light reflected from the sample will be converted into CIELAB color space, also referred to as L*a*b*, thereby determining TP of the composite when measuring samples on black and white backgrounds (3).

Because of the popularity of resin composite materials and the prospects of digital dentistry in cosmetic restoration, studying the relationship between the translucency of composites and the accuracy of the intraoral-scanner (IOS) image may help dentist’s treatment more accurate, bringing optimal results to the patient (4).

A successful restoration depends on the accuracy of the digital impressions. Accuracy is defined as “trueness” and “precision.” According to ISO 5725 standards, “trueness” is the degree to which the true or accepted reference value and the arithmetic mean of a large number of test results agree. The term “precision” describes how closely test results agree with one another (5-8). Our earlier research discovered that composite translucency has an impact on the accuracy of IOS impressions in core build-up restoration of single anterior incisor models qualitatively (4). Here the present study was conducted to clarify whether different translucencies of composite materials affect the accuracy of IOS devices quantitatively, in order to ensure the accuracy of digital impression-taking and bring the best treatment results. We evaluated the correlation between the translucency of dental composite plates of different thicknesses and the accuracy of images recorded and reconstructed by the IOS system.

## Material and Methods

-Specimen preparation

**Figure 1:**
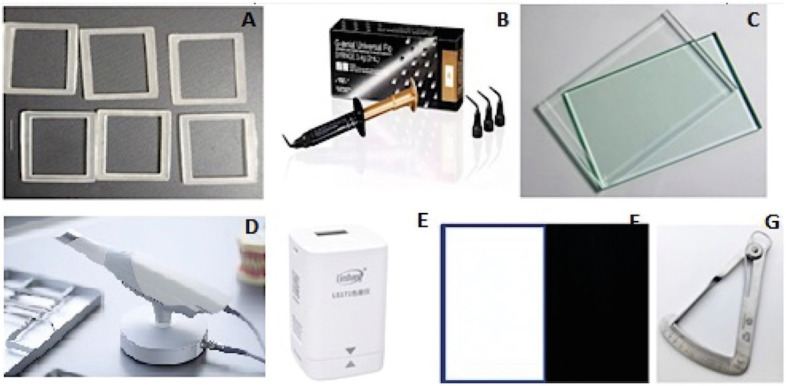
Research materials. A. 3D printed mold. B. GC injectable JE composite. C. Glass slides. D. IOS device. E. Colorimeter. F. Black and white backgrounds. G. Dental caliper.

**Figure 2:**
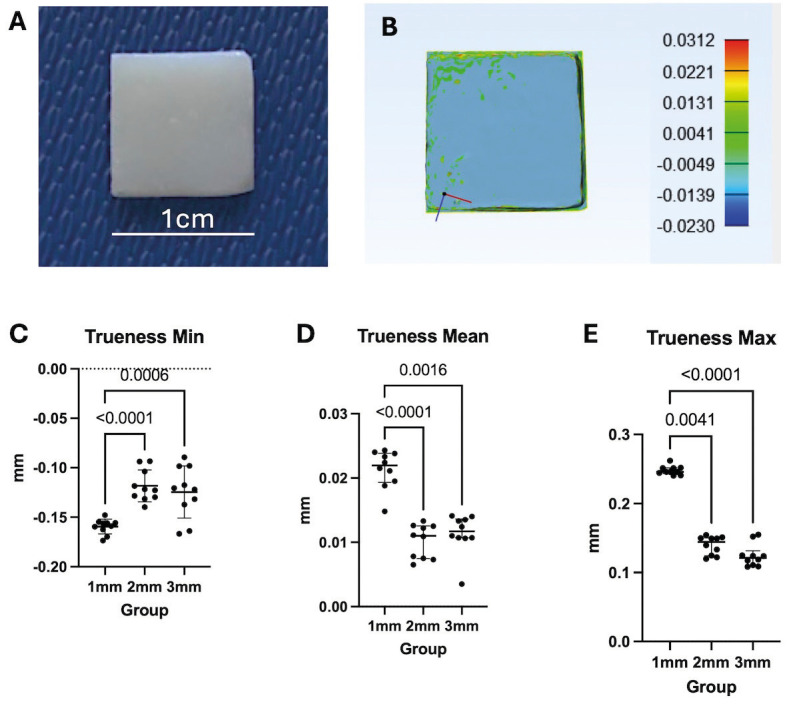
The trueness of IOS was affected by resin composite thicknesses. A. Composite plate. B. The result of superimposition reference scan and experimental scan. The scale reduction was mainly located at the edge of the plate (deviation ladder in mm). Trueness Min (C), Mean (D) and Max (E) deviations of three thicknesses. The 1mm group showed the most deviation. * *p*<0.05, ** *p*<0.01, *** *p*<0.001, **** *p*<0.0001.

Our in-vitro experimental study was performed on 3 G-ænial Universal Flo JE composite (GC, Japan) plates at 3 different thicknesses (1-2-3mm). Composite plates were prepared using 3-printed molds (10x10x1/2/3mm) and glass slides by injecTable method and light curing. Plate thickness was confirmed by dental caliper at 13 points around the plates (1 in the center, 4 in the corners and 8 in the midpoints of the first 5 points) (Fig. 1A-C, G, Fig. 2A). The accepTable thickness deviation threshold was ±0.05mm.

-Scanning procedure

Each plate was scanned 10 times by an IOS device (Trios3, 3Shape, Denmark) for experimental scan data. The scanning was done by a well-trained professional as in our previous studies (4,9). To acquire the reference data, plates were TiO2 powder-coated and scanned by a lab scanner (3Shape E1) (Fig. 1D) (10). All IOS scans and the reference scans were saved and exported in standard tessellation language (STL) format.

-Superimposition procedure

For 3D superimposition and measurement, 3-Matic Research (Materialise N.V., version 13.0, Technologielaan 15, 3001 Leuven, Belgium) was applied. A two-step alignment procedure was used to superimpose each pair of scan data. Using 3 reference points in the corners of the plates, N-point registration was first carried out. After that, global-registration was used to reduce the minimal gap between the two models. The part-comparison was run once the superimposition was complete to display the difference between the two scans. The 3D picture displayed the deviation results along with a color scale, where the blue region represents the positive deviation (experiment > reference) and the red area represents the negative departure (experiment < reference). Each IOS scan was imposed with the reference scan (n=10) to compute the trueness. Each IOS scan file was aligned with every other IOS scan in the same group (C210 =45) in order to compute the precision. These parameters were recorded by software including Min, Mean and Max (µm) deviations between two scans.

-Translucency measurement

The TP of composite plates was measured using LS170 V2.0 colorimeter (Linshang, China) (Fig. 1E,F). Composite plates were placed on black (L* = 3.1, a* = 0.7 and b* = 2.4) and then white (L* = 94.2, a* = 1.3 and b* = 1.7) background panels to measure the color parameters (Lab*) 10 times for each plate (2). L* indicates lightness (0 to 100), and a* and b* indicate levels of red (+a*), green (-a*), yellow (+b*), and blue (-b*) (-60 to +60) (11). From L*, a*, b* values at back (B) and white (W) background, we calculated the TP values according to the CIELAB formula (12): TP = ((L*B- L*W)2 + (a*B- a*W)2 + (b*B- b*W)2)1/2.

-Statistical analysis

JASP (version 0.16, University of Amsterdam, Amsterdam, The Netherlands) was used to conduct the statistical analysis. The median and interquartile, or mean ± standard deviation (SD), were used to depict data. The Shapiro-Wilk test was used to verify normality, and Levene’s test was used to verify variance equality. One-way ANOVA and Tukey’s post hoc test were used to examine data with a normal distribution between groups. Dunn’s post hoc test and the nonparametric Kruskal-Wallis test were used to compare data that did not follow a normal distribution. The correlation between TP and trueness was assessed using Pearson’s r and Spearman’s rho tests. P-values less than 0.05 were regarded as statistically significant.

## Results

Effect of resin composite translucency on IOS trueness

The composite plate scan images were superimposed, as seen in Fig. 2B. The outcome showed that the experiment data’s scale reduced from yellow to red areas when compared to the reference data (experiment < reference). This decrease was more pronounced in the plate border. Table 1, Figure 2C shows that the 1mm group had the lowest significant min deviation (-0.16mm), while the 2mm and 3mm groups obtained the same value (-0.12mm). The 1mm group had the largest scale reduction (0.02mm), followed by the 2mm and 3mm groups (same value 0.01mm). This order is consistent with the mean value of trueness (Table 1, Fig. 2D). The 1mm group (0.25mm) exhibited the most distortion, followed by the 2mm group (0.14mm) and the 3mm group (0.12mm) ( Table 1, Fig. 2E). The findings showed that the 1mm group had the lowest trueness, while the 2mm and 3mm thickness plates had the highest trueness. Composite plates with a thickness of 1 mm generally displayed the lowest trueness. Effect of resin composite translucency on IOS precision

**Figure 3:**
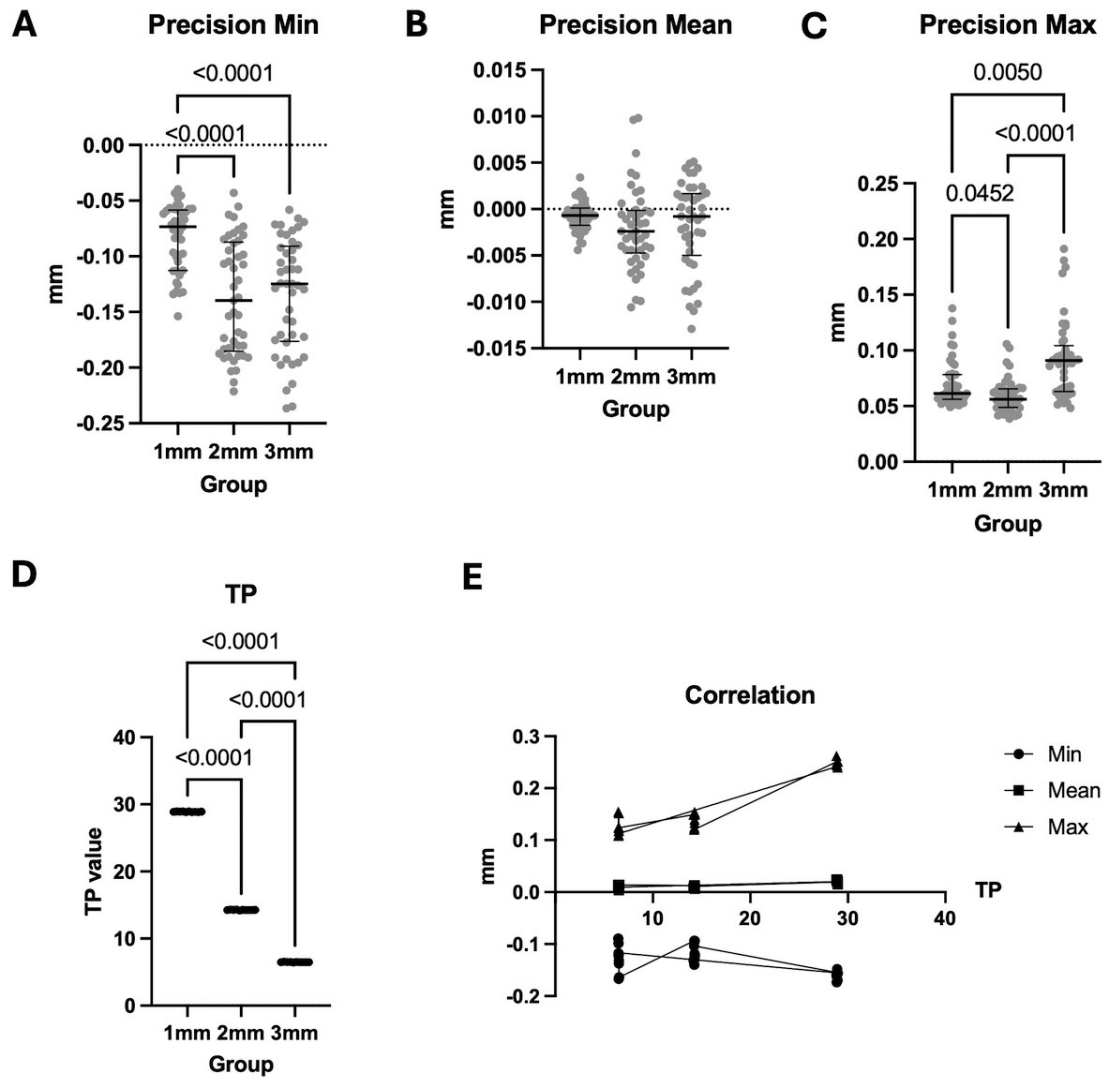
The precision and translucency of composite plates in three thicknesses. Precision Min (A), Mean (B) and Max (C) deviations of three thicknesses. D. Translucency value (TP) of three thicknesses. The 1mm group showed the highest TP. E. Correlation of translucency value and trueness min, mean and max of three thicknesses. * *p*<0.05, ** *p*<0.01, *** *p*<0.001, **** *p*<0.0001.

 Table 2 and Fig. 3A-C displayed the precision of the IOS scan data, with the same scan data in each group, the lower the precision value. The accuracy values’ min and max deviations from 1mm to 3mm groups were often distorted. For the 1mm, 2mm, and 3mm thickness groups, the mean precision of all IOS scan data was extremely low: -0.000731mm (-7.31µm), -0.0021mm (-2.1µm), and -0.00171mm (-1.71µm). The precision measurement’s mean deviation did not, however, differ significantly throughout the three groups. These findings suggested that every thickness performed well in terms of precision.

-Resin composite translucency value correlates to IOS trueness

The colorimeter detects the L*a*b* values of 3 groups followed by TP calculation (Fig. 3D and  Table 3). The results showed that 1mm composite plate expressed the highest TP value, followed by 2mm and 3mm groups (28.90, 14.26 and 6.49 respectively). Correlation analysis of composite plates’ TP and IOS trueness revealed that TP had a high negative correlation to min deviation (Pearson’s r -0.64 and Spearman’s rho -0.57). Meanwhile, TP and mean, max deviation had very high positive correlation (Pearson’s r 0.79 and Spearman’s rho 0.56 for TP vs. mean deviation, and Pearson’s r 0.95 and Spearman’s rho 0.79 for TP vs. max deviation) (Fig. 3E and  Table 4). No correlation was found between TP and precision deviation (data not shown). In brief, the thinner composite, the higher translucency and TP were correlated to IOS trueness of composite plates).

## Discussion

This is the first study to investigate the correlation of TP and IOS accuracy on composite material. Our findings demonstrated that the translucency of resin composites clearly had an impact on Trios3 IOS accuracy. Mean trueness deviations of all groups were 10-20µm indicating the high trueness of this IOS device in comparison to other systems as previous studies on composite materials (13-15). The digital workflow process necessitates the utmost accuracy in order to showcase the exceptional benefit of digital over traditional methods. Digital impression data collection is the first stage that determines if the rest technique is successful (16). Considering the deviations of 120μm (0.12mm) as clinical acceptability of cement gap for CAD/CAM crown (17-20), mean deviations of trueness of 3 thicknesses were passed resulting in good internal fitness and/or marginal gap for the first step of CAD/CAM process: impression. However, resin composite translucency influences both surface noise and max trueness value (up to 0.25mm in the 1mm group). Surface noise may be manifested in the irregular scan data surface and the appearance of larger spots relative to the real surface. The findings demonstrated that noise increases with composite translucency where the composite’s scattering and reflecting properties could be the cause of the distorted scan image.

The repeatability of the scan data is represented by the precision value. The precision mean value that is smaller and more convergent indicates higher data reliability. In the present study, the 3 groups’ mean deviations for the accuracy measurement did not differ substantially from one another. These results implied that all thicknesses had good precise performance. Min and max deviations of precision performed converse from mean deviation. However, the deviation was around the clinical acceptability threshold (120μm) which did not affect the final result. This discrepancy between trueness and precision indicated data noise that needed to be further investigated (4).

By a colorimeter, L*a*b* values of 3 groups were measured and TP was calculated. We investigated G-ænial Universal Flo composite - a nanohybrid flowable composite at JE shade to determine how translucency was changed with the thickness of the material. The results elucidated that the thinner the composite plate, the higher TP. In detail, the 1mm plate has 28.9 TP being similar to SDII 1mm enamel A3 composite discs (28.02-29.32 TP) from a previous study (2). At 2mm thickness, the TP was 14.26 and comparable to BF 1mm nanohybrid A1 (14.22 TP), ES 1mm suprananofill A3.5 (14.36) and F3 1mm nanofill A3.5 (14.26 TP) discs (12). Finally, the 3mm thickness plate has 6.49 TP, the same as F2 2mm nanofill A3.5 (6.49 TP) and GD 2mm microhybrid A2 (6.69 TP) discs (12). Significantly TP and thickness of composite were highly correlated to IOS trueness.

This is the first-time data was demonstrated quantitively where the thinner composite, the higher translucency and TP affected strongly IOS trueness of composite). Within the limitations of our in-vitro study, the design of composite plates did not simulate the shape of the tooth used in clinical practice as well as lacked external influencing factors such as light and saliva during scanning and color measurement. In addition, this study prioritizes choosing a monochromatic composite of the outer color, G-ænial Universal Flo JE (bis-GMA free), as a research sample, without a broader survey of other monochrome and multicolor composite groups. We selected the enamel color to clearly observe the effect of thickness on translucency. Further comprehensive studies investigate the influences of all optical properties of resin composite such as color, opacity, translucency, scattering and light absorption on IOS accuracy. There should be also more research done to look into the relationship between IOS accuracy and bis-GMA containing as well as filler types and particle size of resin composite.

In conclusion, composite translucency has an impact on optical impression accuracy. In correlation, the optical impression becomes less accurate the more translucent the composite is. Clinically, it is strongly recommended that practitioners select not only appropriate translucency but also proper thickness of restoration before digital compression regarding both their mechanical, optical and aesthetic qualities.

## Figures and Tables

**Table 1:** Trueness values of composite plates in three thicknesses.

	Group	Mean	SD	25^th^ percentile	Median	75^th^ percentile
MIN	1	-0.16	7.29×10^-3^	-0.16	-0.16	-0.16
MIN	2	-0.12	0.02	-0.13	-0.12	-0.11
MIN	3	-0.12	0.03	-0.14	-0.12	-0.10
MEAN	1	0.02	2.97×10^-3^	0.02	0.02	0.02
MEAN	2	0.01	2.61×10^-3^	7.58×10^-3^	0.01	0.01
MEAN	3	0.01	3.11×10^-3^	0.01	0.01	0.01
MAX	1	0.25	6.48×10^-3^	0.24	0.25	0.25
MAX	2	0.14	0.01	0.13	0.14	0.15
MAX	3	0.12	0.02	0.11	0.12	0.12

**Table 2:** Precision values of composite plates in three thicknesses.

	Group	Mean	SD	25^th^ percentile	Median	75^th^ percentile
MIN	1	-0.08	0.03	-0.11	-0.07	-0.06
MIN	2	-0.14	0.05	-0.18	-0.14	-0.09
MIN	3	-0.13	0.05	-0.18	-0.12	-0.09
MEAN	1	-7.31×10^-4^	1.57×10^-3^	-1.70×10^-3^	-7.00×10^-4^	1.00×10^-4^
MEAN	2	-2.10×10^-3^	4.46×10^-3^	-4.40×10^-3^	-2.40×10^-3^	-2.00×10^-4^
MEAN	3	-1.71×10^-3^	4.77×10^-3^	-4.60×10^-3^	-8.00×10^-4^	1.60×10^-3^
MAX	1	0.07	0.02	0.06	0.06	0.08
MAX	2	0.06	0.02	0.05	0.06	0.07
MAX	3	0.09	0.04	0.06	0.09	0.10

**Table 3:** L*a*b* at black (B) and white (W) backhround and translucency parameter (TP) of composite plates in three thicknesses.

Group	L^*^_B_±SD	a^*^_B_±SD	b^*^_B_±SD	L^*^_W_±SD	a^*^_W_±SD	b^*^_W_±SD	TP ±SD
1	94.576±0.06	1.341±0.01	-1.145±0.02	121.445±0.06	3.352±0.02	9.302±0.04	28.90±0.04
2	84.483±0.02	0.474±0.01	-7.596±0.02	97.143±0.03	1.019±0.02	-1.048±0.02	14.26±0.04
3	65.906±0.03	-3.178±0.01	-4.773±0.02	70.896±0.03	-2.879±0.02	-0.634±0.03	6.49±0.03

**Table 4:** Correlation of translucency parameter (TP) and min, mean and max deviations of trueness in three thicknesses.

Test	MIN	MEAN	MAX
Pearson's r	-0.64	0.79	0.95
p-value	1.54×10^−4^	2.19×10^−7^	1.38×10^−15^
Spearman's rho	-0.57	0.56	0.79
p-value	1.06×10^−3^	1.32×10^−3^	1.64×10^−7^

## Data Availability

The datasets used and/or analyzed during the current study are available from the corresponding author.
